# Psoriasis and Canine Exposure Leading to Recurrent Prosthetic Joint Infection: A Case Report

**DOI:** 10.7759/cureus.90595

**Published:** 2025-08-20

**Authors:** Yacine Majid, Maxime Maton, Frederic De Vuyst, Pierre-Yves Descamps

**Affiliations:** 1 Department of Orthopedic Surgery, Université Libre de Bruxelles, Brussels, BEL; 2 Department of Orthopedic Surgery, Centre Hospitalier Régional de la Haute Senne, Soignies, BEL

**Keywords:** canine, prosthetic joint infection, psoriasis, total hip arthroplasty, two-stage revision, zoonotic transmission

## Abstract

Prosthetic joint infection (PJI) is a severe complication following total hip arthroplasty (THA), often requiring extensive medical and surgical intervention. This report presents a case of recurrent PJI associated with an untreated psoriatic lesion and habitual canine contact, highlighting an unusual yet significant vector for microbial transmission.

A 51-year-old male patient underwent a primary total hip replacement in January 2024. Three weeks postoperatively, he developed a PJI caused by *Streptococcus agalactiae*. Despite a two-stage revision arthroplasty, recurrent infections were noted over the following months, with cultures later identifying *Staphylococcus aureus* and *Staphylococcus xylosus*. During hospitalization, the patient disclosed a longstanding psoriatic lesion on his knee, which he frequently scratched and allowed his dog to lick. Dermatological consultation confirmed psoriasis, and the association between his skin condition and bacterial transmission from his pet was suspected to contribute to the persistent infection. The patient demonstrated clinical and biological improvement following prosthetic revision and targeted dermatological management.

This case emphasizes the importance of dermatological evaluation in patients undergoing joint replacement, particularly those with chronic inflammatory skin diseases such as psoriasis. Additionally, it underscores the need for thorough patient education on zoonotic bacterial risks. Recognizing the role of dermatological integrity and pet-associated bacterial transmission in PJI can enhance preoperative risk assessment and postoperative care strategies.

## Introduction

Prosthetic joint infection (PJI) is a serious complication following total hip arthroplasty (THA), often leading to significant morbidity and requiring extensive surgical and medical interventions [1,2]. Despite advancements in surgical techniques and infection control protocols, recurrent PJI remains a significant concern.

Various risk factors have been identified in the development of PJIs, including rheumatologic disease, obesity, coagulopathy, and preoperative anemia [3]. Furthermore, external factors, such as environmental exposures and hygiene practices, may further modulate the risk of infection in these patients. Among the most frequently implicated microorganisms in PJI are *Staphylococcus aureus*, coagulase-negative staphylococci, and *Streptococcus *species [4]. We present a case of recurrent PJI in a patient with an untreated psoriasis lesion, where habitual canine contact likely served as a vector for microbial transmission.

## Case presentation

**Figure 1:**
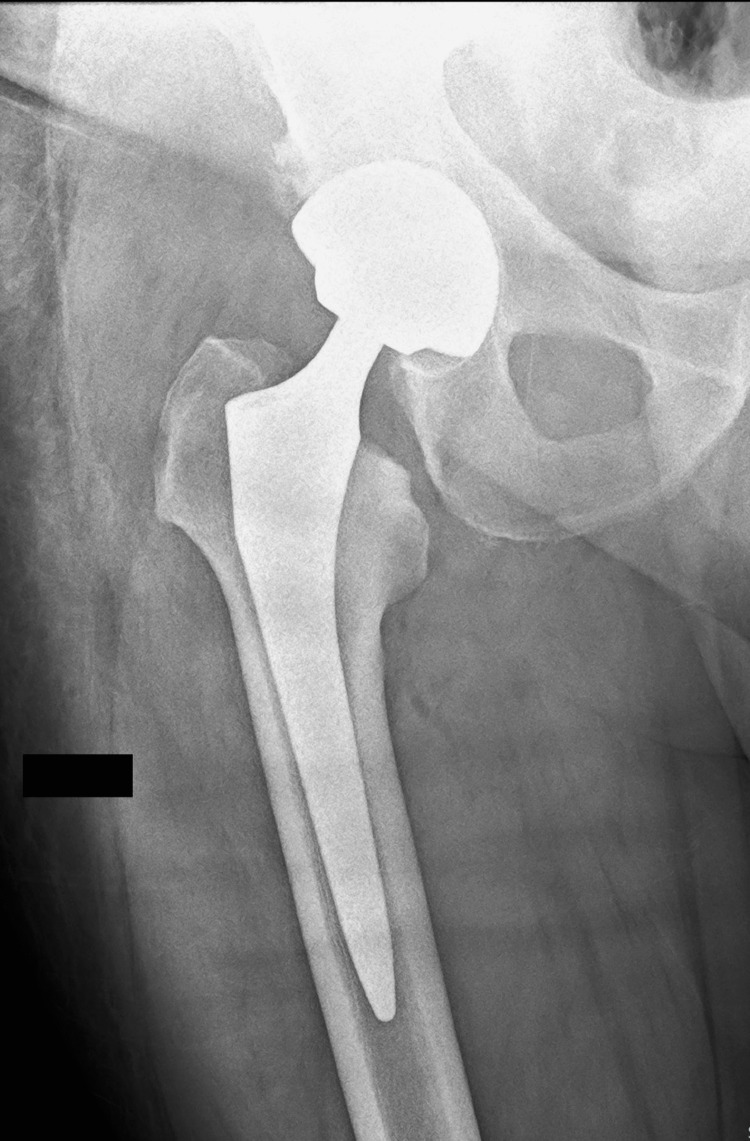
Radiograph of the initial total hip arthroplasty showing the uncemented prosthesis in place after surgery.

A 51-year-old patient underwent an uncemented primary total hip replacement in January 2024 by direct lateral approach (Hardinge). After five days, the patient was discharged from the hospital without any complications (Figure 1).

Three weeks after the intervention, the patient presented with marked redness around the surgical scar, accompanied by a purulent discharge from the wound and a temperature of 38.7°C. Biologically, the patient presented with an elevated CRP of 226 mg/dL as well as an elevated white blood cell count of 18,610/microliter (Table 1).

**Table 1:** Inflammatory markers at different time points during revision hip arthroplasty

Time point	CRP (mg/L)	WBC (×10⁹/L)	Reference range: CRP	Reference range: WBC
Three weeks after the initial intervention	226	18.61	< 5	4.0 – 11.0
One month after the first stage of revision surgery	27	8.46	< 5	4.0 – 11.0
One month after definitive prosthesis insertion (second stage)	213	13.75	< 5	4.0 – 11.0
Two weeks after the second revision	29	9.67	< 5	4.0 – 11.0

In February 2024, debridement of the infected tissues and irrigation were performed in the operating room, and the acetabular liner and the femoral head of the hip prosthesis were changed. Samples taken during the operation revealed the presence of *Streptococcus agalactiae*. In accordance with the antibiogram results, the patient was treated with two grams of amoxicillin and clavulanate three times a day intravenously, followed by a six-month course orally. Clinical and biological improvement in the patient's symptoms was observed during the usual postoperative consultations, one and three months after the operation.

At the beginning of August 2024, the patient returned to our institution, complaining of groin pain, heat, and redness around the surgical scar. He reported a temperature of 40°C the previous day and 38°C on the day of his consultation. Clinical examination also revealed an erythematous-keratotic lesion on his right knee, which had never been examined by a dermatologist. Given the strong suspicion of a recurrence of infection, antibiotic therapy with intravenous amoxicillin and clavulanate was started on the 10^th^ of August after discussing the matter with the infectious disease team and in accordance with the antibiogram results. The antibiotherapy was continued until the end of August 2024.

**Figure 2:**
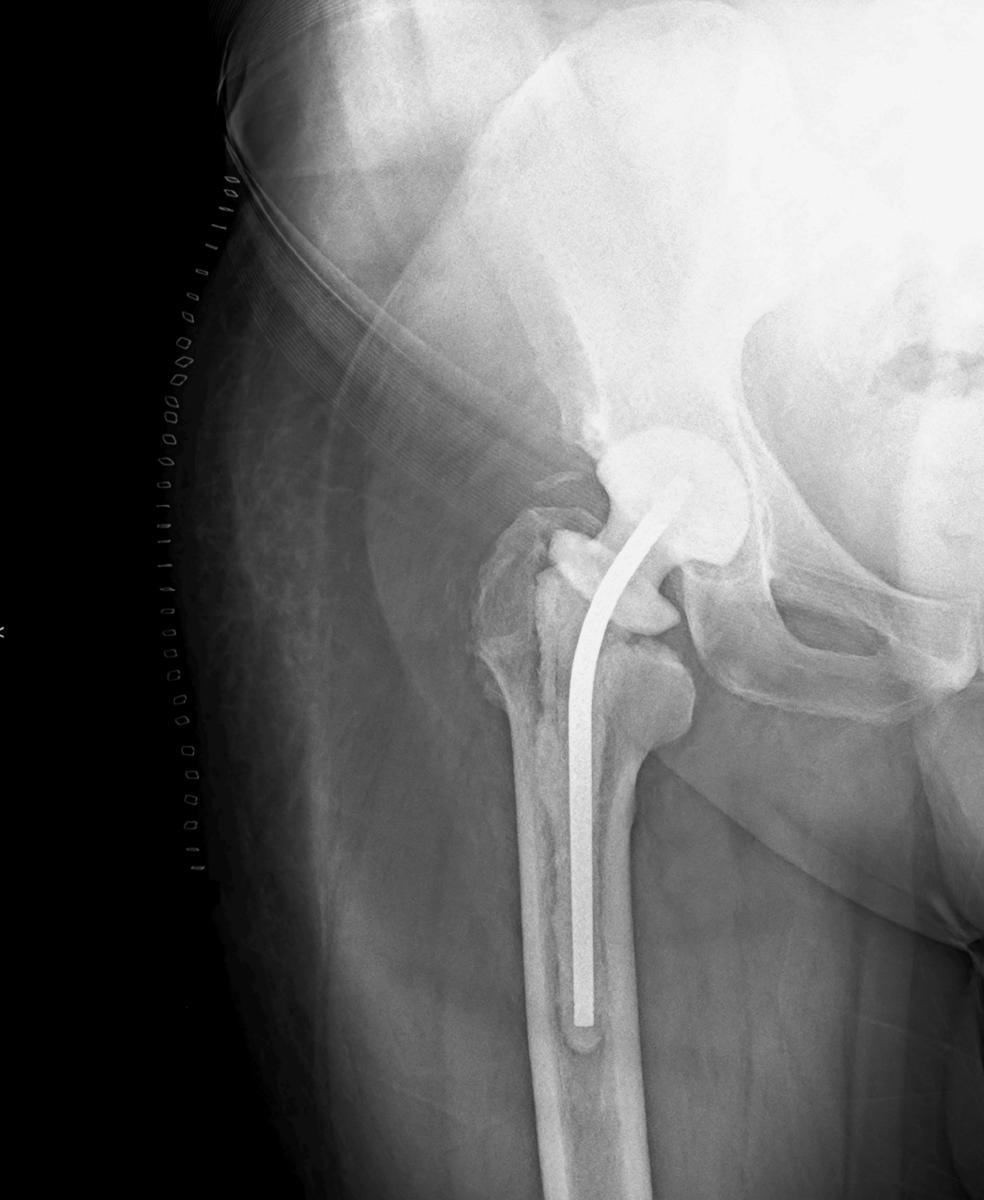
Radiograph of the antibiotic-loaded spacer inserted during the two-stage revision procedure.

Biological deterioration was observed, which motivated the decision to do a two-stage revision prosthesis. In August 2024, he underwent an explant of the prosthesis, and a vancomycin antibiotic-loaded spacer was placed (Figure 2). Samples taken during this operation revealed the presence of *Staphylococcus aureus* and *Staphylococcus xylosus*. Based on the antibiogram, the patient was then placed on moxifloxacin 400 mg daily and rifampicin 600 mg once daily for six weeks.

**Figure 3:**
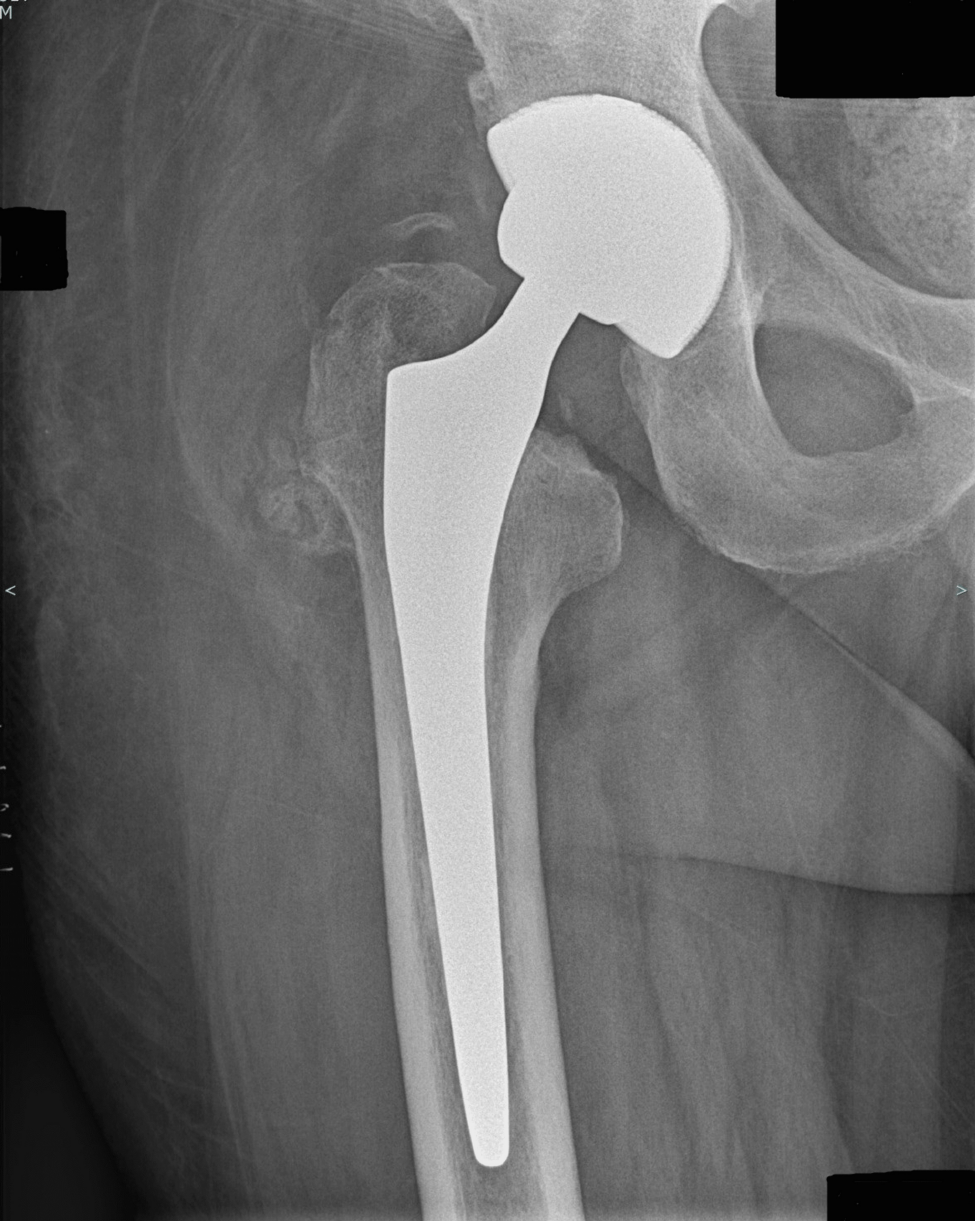
Radiograph of the revision arthroplasty showing the new hip prosthesis after the second stage of surgery.

At the postoperative follow-up, one month after the first step of the revision surgery, CRP had fallen to 27 mg/dL (Table 1), and passive mobilization of the hip was painless. Six weeks after the operation, X-rays showed that there had been no secondary displacement of the spacer. In October 2024, the spacer was removed, and the definitive hip prosthesis was inserted (Figure 3).

In November 2024, the patient returned complaining of pain in his right hip for three days, as well as a purulent discharge from the surgical scar for two days. He also reported a fever, measured at 39°C the previous day. Clinical examination revealed an erythematous scar. Blood tests revealed a CRP of 213 mg/dL and an increase in white blood cell count to 13,750/microliter (Table 1). The following day, debridement of the infected tissues and irrigation were done, as well as a change of the acetabular liner and the femoral head. Intraoperative swabs again showed the presence of *Streptococcus agalactiae*, the same bacterium identified in the initial infection in February 2024. Antibiotic therapy was initiated with amoxiclav, at a dose of one gram four times a day, combined with moxifloxacin 400 milligrams once a day, as advised by the infectiology team.

**Figure 4:**
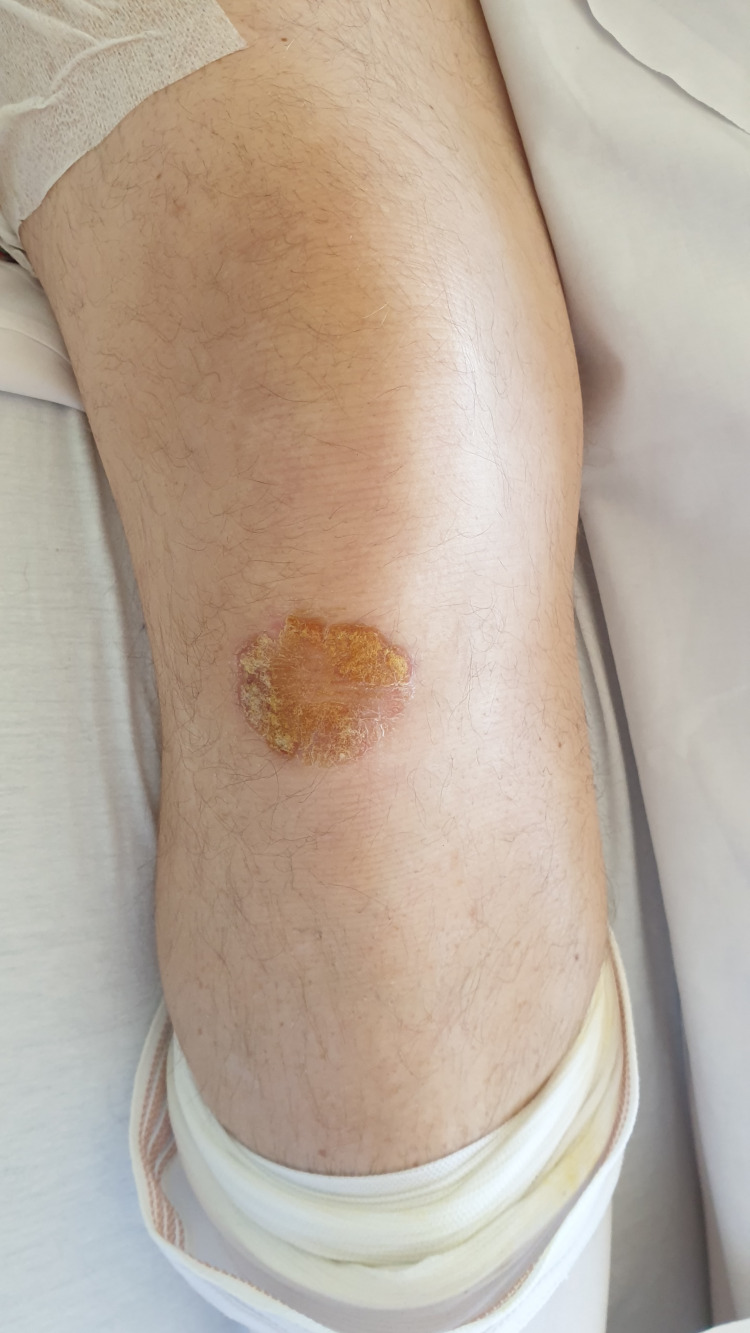
Clinical image of the psoriatic lesion on the patient’s right knee.

It was during this hospitalization that the patient revealed an itchy lesion on his right knee, causing him to scratch himself frequently with his fingers. He also told our team that he made his dog lick this same lesion as a gesture that temporarily soothes his itch. According to the patient, this behavior was present before and after the initial THA in January 2024. A dermatological consult was requested, and a diagnosis of psoriasis was confirmed (Figure 4). The dermatologist recommended appropriate treatment for this dermatosis, which was thought to play a role in the onset and recurrence of prosthetic infections as well as the dog's licking of the lesion.

In December 2024, the prosthesis was replaced due to persistence of the infection. The decision was made to proceed with a one-stage revision arthroplasty. The femoral stem was fixed with cement containing antibiotics (gentamicin + vancomycin).

The patient subsequently had a good clinical and biological outcome during his hospital stay. We also recommended that he avoid any contact between his dog's mouth and the psoriasis lesion. Dermatological management of the lesion was put in place.

**Figure 5:**
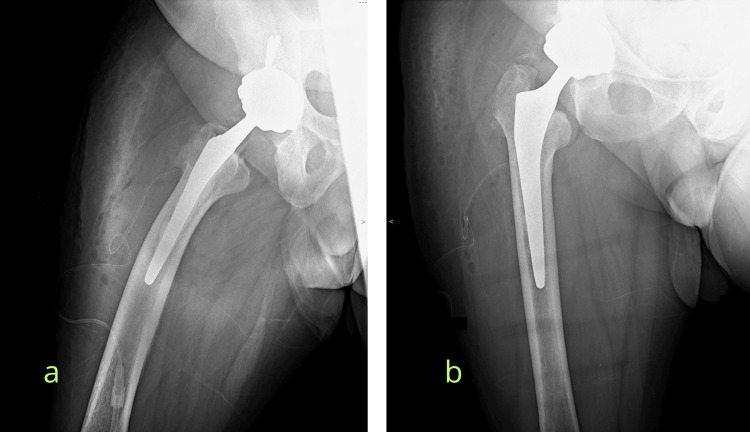
Final radiographs after the second revision. Lateral (a) and anteroposterior (b) radiographs of the right hip following the one-stage revision total hip arthroplasty.

At the postoperative consultation, one month after surgery, the patient showed no signs of reinfection or instability of his prosthesis (Figure 5).

## Discussion

Infection in THA is a considerable concern among orthopedic surgeons. The incidence of PJI following primary total hip replacement ranges around 0.61% [5]. However, the infection rate is significantly higher following revision surgery, with an estimated incidence of 13.1% for one-stage revisions and 10.4% for two-stage revisions [6].

This case highlights the significant role of psoriasis as a predisposing factor for PJI. Psoriasis is a chronic inflammatory skin condition that has been associated with an increased risk of surgical site infections (SSIs) and PJIs. Gold PA et al. [7] showed in a 10,727 total knee arthroplasty (TKA) patient database an increased associated risk of deep SSIs and wound complications compared to non-psoriasis patients. Drancourt et al. [8] found evidence in a case study that psoriasis was a risk factor for hip prosthesis infection.

The impaired skin barrier function and immune dysregulation caused by psoriasis provide an entry point for opportunistic pathogens, increasing the risk of deep-seated infections [9]. In this case, the patient had an untreated psoriatic lesion on his right knee, which he frequently scratched with his hands, further compromising his skin integrity. The repeated trauma to the psoriatic lesion likely facilitated microbial invasion, leading to persistent and recurrent infections of the prosthetic joint despite a thoroughly made two-stage revision hip arthroplasty.

Another remarkable aspect of this case is the patient’s habit of allowing his dog to lick his psoriatic lesion, which likely served as a vector for bacterial transmission. Direct contact with animal saliva has been linked to the transmission of pathogenic bacteria, which can cause serious infections, including PJIs. Lam PW et al. [10] did a literature review and found 32 documented cases of PJI by *Pasteurella multocida*, a Gram-negative coccobacillus that is part of the normal oral flora of animals, including domestic cats and dogs; almost all patients had a history of animal contact, with 26 cases of soft tissue injury as a result. Twenty-two of the cases involved cats, while 10 cases involved dogs.

Dog saliva harbors a variety of potentially harmful bacteria, including *Streptococcus agalactiae* and *Staphylococcus aureus*, both of which were identified in the patient’s prosthetic infections. Although *Staphylococcus aureus* and Streptococcus agalactiae are not the germs most frequently found in dog saliva, they can be present [11,12].

In this case, the patient’s repeated exposure to canine saliva may have contributed to both the initial and recurrent infections of his prosthetic joint. The recognition of this unusual risk factor underscores the need for comprehensive patient education on infection prevention. Patients with psoriasis or other chronic skin conditions should be advised to avoid direct pet saliva contact with affected areas, practice proper wound hygiene, and seek timely dermatological care.

## Conclusions

This case underscores the multifactorial risks contributing to PJI, particularly the interplay between chronic skin conditions like psoriasis and zoonotic bacterial exposure. Recognizing psoriasis as a significant risk factor for PJI highlights the need for proactive dermatological management in arthroplasty patients with dermatological conditions. Clinicians should incorporate these considerations into preoperative risk assessments and postoperative care strategies to optimize patient outcomes.
